# Effects of Dietary N-Carbamylglutamate Addition with Two Different Levels of Crude Protein (Low and High) on the Growth, Nitrogen Retention, Amino Acids in the Carcass, Blood Health, Gut Bacteria, and Meat Quality of Finishing Pigs

**DOI:** 10.3390/ani16162484

**Published:** 2026-08-10

**Authors:** Usman Kayode Kolawole, Kye Jin Lee, Youngjong Han, Minjeong Kim, Kyudong Han, Dae-Kyung Kang, In Ho Kim

**Affiliations:** 1 Department of Animal Biotechnology, Dankook University, Cheonan 31116, Republic of Korea; kolawoleusman@rocketmail.com (U.K.K.);; 2 Smart Animal Bio Institute, Dankook University, Cheonan 31116, Republic of Korea; 3 Department of Microbiology, College of Bio-Convergence, Dankook University, Cheonan 31116, Republic of Korea; 4 Departmentof Bioconvergence Engineering, Dankook University, Yonginsi 16890, Republic of Korea

**Keywords:** crude protein density, N-carbamylglutamate (NCG), finishing pigs, performance

## Abstract

This experiment was performed to investigate the effect of two different CP levels, with or without NCG supplementation, on finishing pigs’ performance and meat quality. A total of 200 pigs ([Yorkshire × Landrace] × Duroc) with an initial average body weight (BW) of 50.06 ± 1.34 kg were randomly assigned to one of four treatments for 70 days. There were four treatment groups with ten replicates and five pigs (three males and two females) per pen. The dietary treatment includes low-protein NCG−, 13% CP (LP NCG−); low-protein NCG +, 13% CP + NCG 0.1% (1 kgton) (LP NCG+); high-protein NCG−, 15% CP (HP NCG−); high-protein NCG+, 15% CP + NCG 0.1% (1 kg/ton) (HP NCG+). After 6 weeks of experimentation on CP effect, blood urea nitrogen, total protein, glucose, and albumin levels in pigs fed LP NCG− diets were significantly lower (*p* < 0.05), while in terms of the effect of NCG on creatinine level, the levels in the HP NCG+ diet were significantly lower than those in LP NCG+. Dry matter intake and nitrogen intake in HP NCG− diets were significantly higher than in LP NCG−. Loin muscle area (LMA) and cooking loss in LP NCG+ diets were significantly higher than in HP NCG+. Isoleucine, histidine, lysine, alanine, and tyrosine showed higher values in HP NCG−diets than in LP NCG−. LP NCG+ feeds improved species diversity and abundance of *Firmicutes*, LP NCG− of *Lactobacillus*, and HP NCG+ of *Bacilli*. Thus, low dietary protein can be beneficial for nitrogen retention, blood profile, and carcass amino acids, and NCG supplementation at 0.1% for gut health and meat quality.

## Simple Summary

Lowering the dietary crude protein (CP) content by 1–4% in finishing pigs, when appropriately paired with restricting amino acids, enhances feed efficiency and decreases nitrogen excretion without negatively affecting performance. Adding 0.05–0.1% N-carbamylglutamate (NCG) to these low-protein diets addresses potential protein deficiencies by promoting arginine synthesis, fostering muscle growth, and improving nitrogen utilization. This study investigated the optimum level of two different crude protein densities (low and high) and dietary N-carbamylglutamate supplementation on finishing pigs’ performance, blood profile, gut health, and meat quality. Low-protein diets (13% CP) improved nitrogen retention, blood parameters (blood urea nitrogen, BUN), and carcass amino acids, while 0.1% NCG supplementation improved gut health, and meat quality in finishing pigs. Lowering protein levels is beneficial for improving nitrogen retention and blood parameters, and supplementing with 0.1% NCG can improve gut health and meat quality. In conclusion, a combination of a low-protein diet (13% CP) and 0.1% NCG supplementation is an effective strategy to improve carcass characteristics, blood parameters, gut health, and nitrogen utilization without detrimental effects on performance in finishing pigs.

## 1. Introduction

Current research provides divergent perspectives on how dietary crude protein levels significantly affect finishing pig performance, leading to a lack of consensus in swine nutrition [1]. For finishing pigs, protein is essential for maintaining health, maximizing muscle deposition, and ensuring economical production; 12% to 14% crude protein is usually needed, as finishing periods are important because pigs gain a lot of weight and primarily build muscle mass [2]. A diet that promotes muscular development must be given in order to optimize growth during this time [3]. In order to achieve optimal growth, pigs also need a balanced ratio of amino acids and sufficient nutrients for body protein synthesis [4]. The completion of muscle growth and the start of active fat formation define the finishing phase, which comes after the growth phase [5]. Pork quality is directly impacted by this stage, which is crucial for the buildup of intramuscular fat [1]. The right amount of nutrients guarantees that pigs get what they require throughout growth; if dietary protein intake surpasses pig needs, then the excess is eliminated in feces or urine, which impacts the environment and the economy of production [6]. Protein is the chemical basis of digestive enzymes, which are specialized proteins that hasten the chemical reactions needed to break down food into nutrients; theoretically, a lack of dietary protein might impact the synthesis and activity of these enzymes, leading to a reduction in the effectiveness of digestion [7].

Arginine, which is the most prevalent nitrogen carrier in tissue proteins, has a wide range of important metabolic and regulatory functions [8]. According to earlier research, arginine may improve growing–finishing pigs’ growth performance [9]. Nevertheless, alternatives to arginine must be investigated given the high expense of adding it directly and the possibility that appropriate dose supplementation could affect the metabolism and absorption of other amino acids [10].

By activating carbamoyl phosphate synthase-1 and pyrroline-5 carboxylate synthase, N-carbamoylglutamate, an analog of metabolically stable N-acetylglutamate (NAG), has shown the ability to increase plasma arginine levels and promote endogenous arginine synthesis [11]. As a structural equivalent of NAG, NCG can stimulate the production of endogenous arginine in the intestine and activate the enzyme that limits the rate of arginine synthesis. According to recent research, NCG is crucial for preserving gut health and enhancing growth performance. Dietary NCG supplementation in a protein-reduced diet may raise the longissimus dorsi muscle area, decrease backfat accumulation, and generate functional pig meat with a high leucine content. The regulating influence on meat quality has been demonstrated in finishing pigs [12]. Nevertheless, the use of NCG in finishing pigs has not received much attention up to this point, and there is little information available regarding safety assessment or the ideal dosage of supplementation [10].

The hypothesis is to investigate the optimal dietary crude protein level and NCG addition on finishing pigs’ performance, blood profile, and gut health. Therefore, this study aimed to observe the effect of two crude protein densities, with or without dietary N-carbamylglutamate supplementation, on growth performance, blood profile, nutrient digestibility, nitrogen (N) retention, noxious gas emissions reduction, backfat thickness (BFT), lean meat percentage (LMP), meat quality, carcass grade, amino acid content, and fecal microbial flora in finishing pigs.

## 2. Materials and Methods

All the procedures used in the experiment were revised and accepted by the Institutional Animal Care and Use Committee of Dankook University, Republic of Korea (Ethical approval No. DK-2-2328, Approved on 15 March 2023).

### 2.1. Source of N-Carbamylglutamate

NCG (white powder, ≥98.1%) was procured from Linzhou Animore Sci. & Tech. Co., Ltd., Anyang, China, and incorporated into the control diet at a dose of 0.1% (1 kg/ton).

### 2.2. Animal Husbandry, Diet, Experimental Design

In a 70-day trial, a total of 200 crossbred finishing pigs ([Yorkshire × Landrace] × Duroc) with an initial average BW of 50.06 ± 1.34 kg were used. The pigs were randomly assigned to one of four dietary treatments based on initial BW and sex, with 10 replicate pens per treatment and 5 pigs (3 barrows and 2 gilts) per pen. The treatments consisted of (1) low-protein diet without NCG (13% CP; LP NCG−), (2) low-protein diet with 0.1% NCG (13% CP; LP NCG+), (3) high-protein diet without NCG (15% CP; HP NCG−), and (4) high-protein diet with 0.1% NCG (15% CP; HP NCG+). Pigs were housed in an environmentally controlled facility with a mechanically ventilated aeration system and slatted plastic floors. During the experimental period, pigs had ad libitum access to feed and water via pen-fitted nipple drinkers and feeders. The experimental diets consisted of decreasing CP content (NC, 13% CP). All diets were formulated to contain similar concentrations of standardized ileal digestible (SID) Lys, Met + Cys, Thr, Trp, and NE as per the recommendations of NRC [13]. The low-crude-protein diet (basal diet—2% CP) contains increased batches of corn, tallow, MDCP, methionine, lysine, tryptophan, and threonine; the same batches of DDGS, limestone, salt, and choline; and reduced soyabean meal (Table 1). The diet formulation in the experiment (Table 1) was corn–soybean meal-based feed mixed following the NRC guidelines for finishing pigs [13]. The other nutrients were maintained to meet or exceed the NRC [13] estimated requirements.

### 2.3. Sampling Collections

#### 2.3.1. Growth Performance

The individual body weight of pigs was measured on day 1, week 5, and the last days of week 10 to calculate the pen-based average daily gain (ADG), and feed intake was checked daily for average daily feed intake (ADFI) calculation. The feed conversion ratio (FCR) was calculated using ADG and ADFI values.

#### 2.3.2. Blood Profiles

For blood collection, 5 mL of blood was collected from the jugular vein using a vacuum tube (Becton Dickinson Vacutainer Systems, Franklin Lakes, NJ, USA) at the end of the experiment (10 weeks) and then centrifuged at 3000 rpm for 15 min, and the obtained serum was automatically collected. Using a biochemical analyzer (HITACHI 747, Japan), BUN, creatinine, total protein, albumin, glucose, triglycerides, and cholesterol were measured.

#### 2.3.3. Nutrient Digestibility

Nutrient digestibility was measured at the end of the experiment (10 weeks) by adding 0.5% chromium oxide (Cr_2_O_3_) as an indicator and feeding for 7 days by rectal massage. The collected fecal samples were dried in an oven at 60 °C for 72 h, then ground in a Willey mill and used for analysis. Cr_2_O_3_ mixed with the general ingredients of the feed and the indicator was analyzed according to the method of AOAC (2000). Chromium levels were measured using a UV absorption spectrophotometric method (UV-1201, Shimadzu, Kyoto, Japan). Using an oxygen bomb calorimeter (Parr, 6400 Instrument Company, Moline, IL, USA), a sample of 2 g of feed and feces was examined. Additionally, the Kjeltec 8600 (Foss Tecator AB, Hoeganaes, Sweden) was used to measure N in order to calculate the protein level.

Formula for ATTD:

Nutrient digestibility (%) = [1 − (Nf × Cd)/(Nd × Cf)] × 100,

where Nf—number of nutrients in feces (% DM), Nd—number of nutrients in the diet (% DM), Cd—chromium content in the diet (% DM), and Cf—chromium content in feces (% DM) [14].

#### 2.3.4. Nitrogen Retention

Total nitrogen (N) contents in the diets and diets were determined using a Kjeltec 8600 analyzer (Foss Tecator AB, Hoeganaes, Sweden) following the AOAC [15] methods. Daily N intake was calculated by multiplying the daily feed intake by the N concentration of the respective diet. Fecal N excretion was derived from the daily dry matter output and fecal N content using the chromic oxide ratio. Daily N retention was calculated by subtracting fecal and urinary N losses (where urinary N was estimated based on historical reference equations for finishing pigs [16]) from the total N intake. The apparent N retention efficiency was calculated using the following equation:

Nitrogen retention (%) = [(N intake − Fecal N) ÷ N intake] × 100

#### 2.3.5. Noxious Gas Emissions

To analyze noxious gas emission in feces, feces excreted for the same amount of time were collected from each treatment group at the end of the experiment (10 weeks). Then, 300 g of fresh feces was placed in a sealed plastic container of 2600 mL, fermented, and stored at room temperature for 7 days, and then NH_3_, H_2_S, methyl mercaptan, acetic acid, and CO_2_ were measured using a complex gas meter (MultiRAE Lite model). It was measured using PGM-6208 (RAE, USA).

#### 2.3.6. Backfat Thickness and LMP

Backfat thickness and meat percentage were measured at the P2 point (6.5 cm left and right from the last rib) using Piglog 105 (Carometec food technology, Denmark) at the end of the experiment (10 weeks).

#### 2.3.7. Meat Quality

The sample (pig meat or pork) used for meat quality analysis was stored in a refrigerator at 4 °C for 24 h after slaughter, and the semi-membranous loin (M. longissimus dorsi) was segmented and used for analysis. Meat color was measured twice for each sample using a Chromameter (Model CR-410, Minolta Co., Japan), and the average value was calculated. At this time, the standard color plate was L* (lightness) = 89.2, a* (redness) = 0.921, and b* (yellowness) = 0.783. Water-holding capacity was measured using the method of Hofmann [9], the area was calculated using a planimeter (X-plan, Ushikata 360d∏, Japan), and the surface area of the meat was divided by the water area. The pH value of meat was measured for all samples after slaughter using a pH meter (Istek, Model 77p). The cross-sectional area of the loin was measured using OHP film, the circumference of the loin was measured, and the area was measured using a planimeter (X-plan, Ushikata 360d∏, Japan). For cooking loss, the weight of the sample was measured by shaping it into a certain shape, then placing it in a polyethylene bag, heating it in a constant-temperature water bath (75 °C) for 30 min, cooling it at room temperature for 30 min, and then measuring the weight of the sample. Sensory evaluation examined meat color (color: 1–5), intramuscular fat (marbling: 1–5), and firmness (firmness: 1–5) of fresh meat based on grading standards and meat color standards based on intramuscular fat content. Storage loss (drip loss): the sample was shaped into a regular shape with a thickness of 2 cm, placed in a polyethylene bag, and stored in a 4 °C refrigerator for 7 days. Weight loss occurring after 1, 3, 5, and 7 days was measured.

#### 2.3.8. Carcass Amino Acids

To measure the amino acid composition in the carcass, the post-slaughter sample was dissolved in a solution of water and ethanol (1:1) at 4 °C for 10 min. After centrifugation for 10 min, 40 μL of the supernatant was labeled with iTRAQ reagent and analyzed using Applied Biosystems 3200 Q TRAP LC/MS/MS equipped with an RP-C18-column.

#### 2.3.9. Fecal Microbial Flora Analysis

Microbial DNA was extracted from pig fecal samples with the EZNA^®^ Soil DNA Kit (Omega Bio-tek, Norcross, GA, USA) following the manufacturer’s instructions. DNA integrity was verified on a 1% agarose gel, and concentration and purity were measured using a NanoDrop 2000 UV–vis spectrophotometer (Thermo Fisher Scientific, Waltham, MA, USA). The highly variable V3–V4 regions of the bacterial 16S rRNA gene were amplified with primers 338F (5′-ACTTACTACGGGGGAGGCAGCAG-3′) and 806R (5′-GGACTACHVGGGTWTCTAAT-3′) on an ABI GeneAmp 9700 PCR thermocycler (Applied Biosystems, Foster City, CA, USA). PCR conditions were initial denaturation at 95 °C for 3 min, 27 cycles of denaturation at 95 °C for 30 s, annealing at 55 °C for 30 s, and extension at 72 °C for 45 s, followed by a final extension at 72 °C for 10 min and termination at 4 °C. PCR products were purified and sequenced on the Illumina MiSeq PE300 or NovaSeq PE250 platforms (Illumina, San Diego, CA, USA) according to the standard protocol of Majorbio Bio-Pharm Technology Co., Ltd. (Shanghai, China). OTUs were clustered at a 97% similarity threshold using UPARSE v7.1, and chimeric sequences were identified and removed. Taxonomic classification of representative OTU sequences was performed with the RDP Classifier v2.2 against the Silva v138 16S rRNA database at a confidence threshold of 0.7. Alpha diversity was assessed in Mothur v1.31.2, and beta diversity was evaluated using Principal Coordinate Analysis (PCoA).

### 2.4. Statistical Analysis

Data were analyzed using a 2 × 2 factorial design with the General Linear Model (GLM) procedure in SAS (version 9.4; SAS Institute Inc., Cary, NC, USA). The statistical model included the main effects of crude protein (CP) level, N-carbamylglutamate (NCG) supplementation, and their interaction. The individual pig was considered the experimental unit. When significant interactions (*p* < 0.05) were detected, Tukey’s honest significant difference (HSD) test was applied for post hoc comparisons. For microbial community analysis, raw sequence counts were normalized to a constant depth. Alpha diversity indices (Chao1 and Shannon) were analyzed using two-way ANOVA after confirming normality with the Shapiro–Wilk test. Relative abundance data were centered log-ratio (CLR)-transformed prior to analysis. Effects of CP, NCG, and their interaction on taxonomic abundance were evaluated using two-way ANOVA. Beta diversity was assessed by PERMANOVA based on Bray–Curtis and UniFrac distance matrices (999 permutations). Differential abundance was determined using LEfSe, with False Discovery Rate (FDR) adjustment (Benjamini–Hochberg procedure) applied to *p*-values. *p*-values < 0.05 were considered statistically significant, while values between 0.05 and 0.10 were interpreted as statistical tendencies.

## 3. Results

### 3.1. Growth Performance

The effect of dietary NCG supplementation in low-protein and high-protein diets at the finishing stage on the growth performance of finishing pigs is shown in Table 2. No interaction was observed between protein level and NCG supplementation on growth performance in finishing pigs. FCR tended to decrease, approaching statistical significance (*p* = 0.0547).

### 3.2. Blood Profiles

The effect of dietary NCG supplementation in low-protein and high-protein diets at the finishing pig stage on the blood profiles of finishing pigs is shown in Table 3. At the end of the experiment (10 weeks), protein level influenced BUN, total protein, glucose, and albumin. In contrast, NCG supplementation specifically affected creatinine. BUN (*p* = 0.0005), total protein (*p* = 0.0013), glucose (*p* < 0.0001), and albumin (*p* = 0.0057) were significantly higher in HP NCG− compared to LP NCG−.

### 3.3. Nutrient Digestibility

The effect of dietary NCG supplementation in low-protein and high-protein diets on the nutrient digestibility of finishing pigs is shown in Table 4. Digestibility of dry matter, nitrogen, and energy did not differ among treatments (*p* > 0.05), indicating that neither protein level nor NCG supplementation altered nutrient utilization.

### 3.4. Nitrogen Retention

The effect of dietary NCG supplementation in low-protein and high-protein diets on the nitrogen content in the feces and urine of finishing pigs is shown in Table 5. At the end of the experiment (10 weeks), no significant difference was observed among treatments (*p* > 0.05). The two different dietary crude protein densities, LP NCG− and HP NCG−, influenced dry matter intake (DM intake, g/d) and nitrogen intake (N intake, g/d). DM intake and N intake decreased with the reduction in dietary protein without NCG. DM intake and N intake in HP NCG− were significantly higher than in LP NCG− (*p* = 0.0410, *p* = 0.0310).

### 3.5. Noxious Gas Emissions

The effect of dietary NCG supplementation in low- and high-protein diets at the finishing stage on noxious gas emissions in the feces of finishing pigs is shown in Table 6. There was no significant difference among the treatment groups in NH_3_, H_2_S, methyl mercaptan, acetic acid, or CO_2_ (*p* > 0.05).

### 3.6. Backfat Thickness and LMP

The effect of dietary NCG supplementation in low-protein and high-protein diets on the backfat thickness and meat yield of finishing pigs is shown in Table 7. There was no significant difference among the treatment groups in backfat thickness or LMP (*p* > 0.05).

### 3.7. Meat Quality

The effect of dietary NCG supplementation in low-protein and high-protein diets on the meat quality characteristics of finishing pigs is shown in Table 8. Dietary NCG supplementation (low-protein NCG+ and high-protein NCG+) influenced LMA and cooking loss. Cooking loss increased in the LP + NCG group compared to HP + NCG (*p* = 0.0004). Although higher cooking loss is generally undesirable, this may reflect differences in muscle water-holding capacity.

### 3.8. Carcass Grade and Backfat Thickness

The effect of dietary NCG supplementation in low-protein and high-protein diets on the carcass grade of finishing pigs is shown in Table 9. At the end of the experiment (10 weeks), there was no significant difference among the treatment groups (*p* > 0.05).

### 3.9. Carcass Amino Acids

The effect of dietary NCG supplementation in low-protein and high-protein diets on the carcass amino acids of finishing pigs is shown in Table 10. At the end of the experiment (10 weeks), significant differences were detected (*p* < 0.05) in isoleucine, histidine, lysine, alanine, and tyrosine between HP NCG− and LP NCG−.

### 3.10. Fecal Microbial Flora

#### 3.10.1. Alpha Diversity

**Figure 1:**
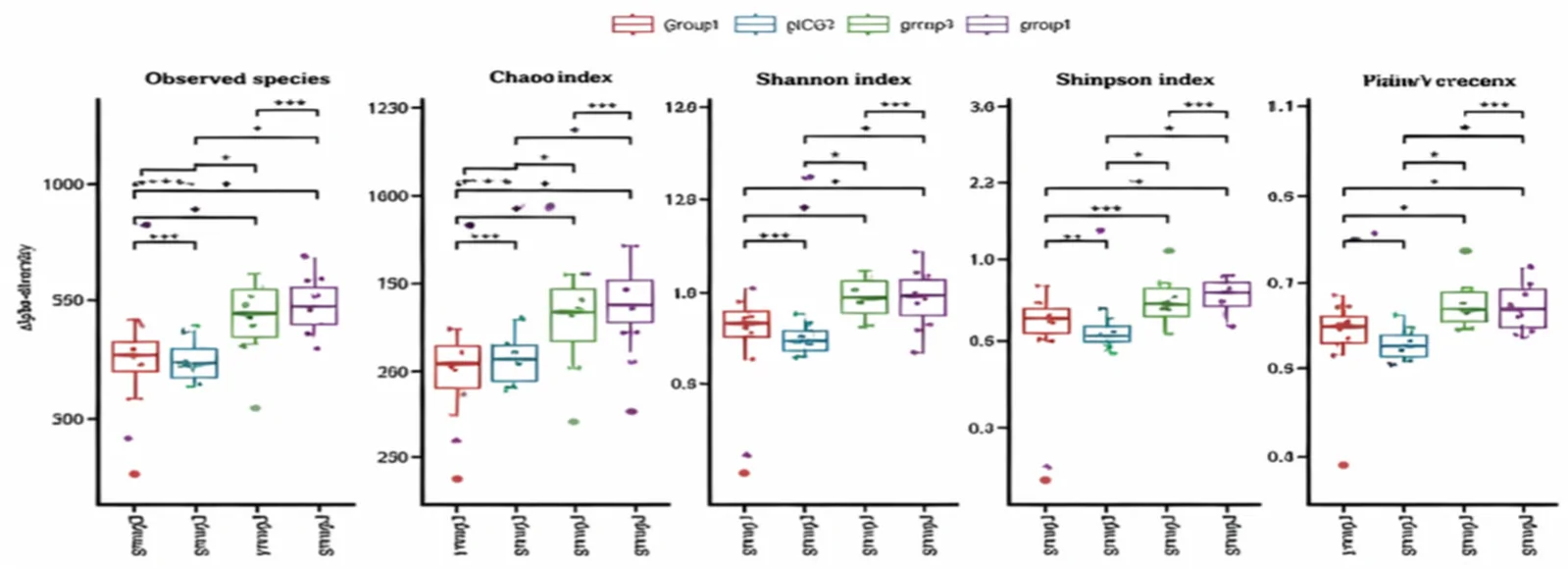
Alpha diversity: Group 1: LP NCG− (13% CP); Group 2: LP NCG+ (13% CP + 0.1% NCG); Group 3: HP NCG− (15% CP); Group 4: HP NCG+ (15% CP + 0.1% NCG). * *p* < 0.05, ** *p* < 0.01 and *** *p* < 0.001.

The diversity and richness of fecal microbial flora were evaluated by the Simpson, Shannon, and Chao1 indices (Figure 1). Alpha diversity was assessed using Chao1, Shannon, Simpson, and Pielou indices (HP NCG− is Group 3 and HP NCG+ is Group 4), as well as low-protein (LP NCG− is Group 1 and LP NCG+ is Group 2) effects on species diversity. In almost every plot, groups with NCG (Groups 2 and 4) show higher median values compared to their respective control groups (Groups 1 and 3). While both high- and low-protein diets showed improvements with NCG, high-protein + NCG (Group 4) generally exhibits the highest overall alpha diversity across the metrics.

#### 3.10.2. Beta Diversity

**Figure 2:**
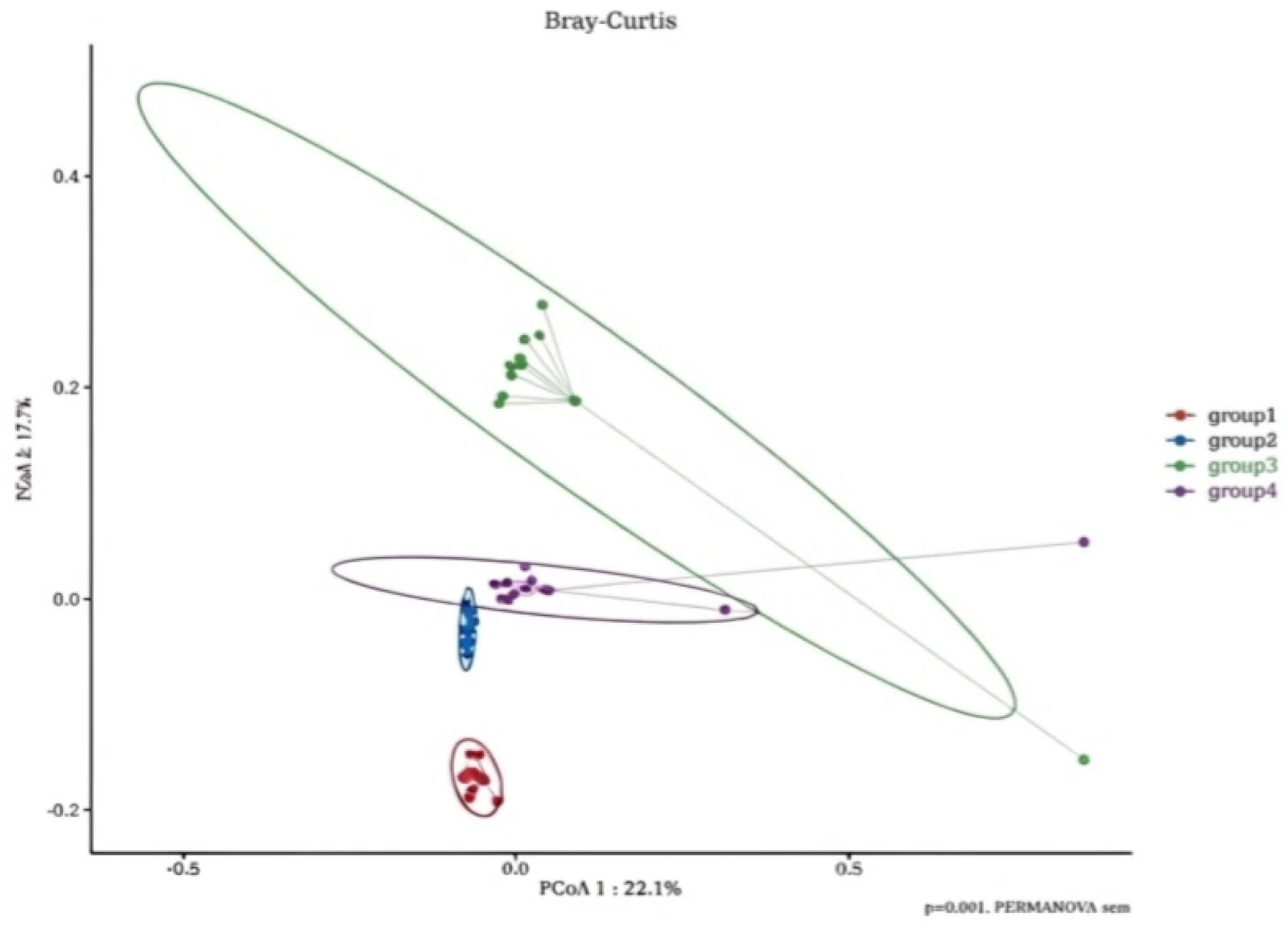
Beta diversity: Group 1: LP NCG− (13% CP); Group 2: LP NCG+ (13% CP + 0.1% NCG); Group 3: HP NCG− (15% CP); Group 4: HP NCG+ (15% CP + 0.1% NCG).

**Figure 3:**
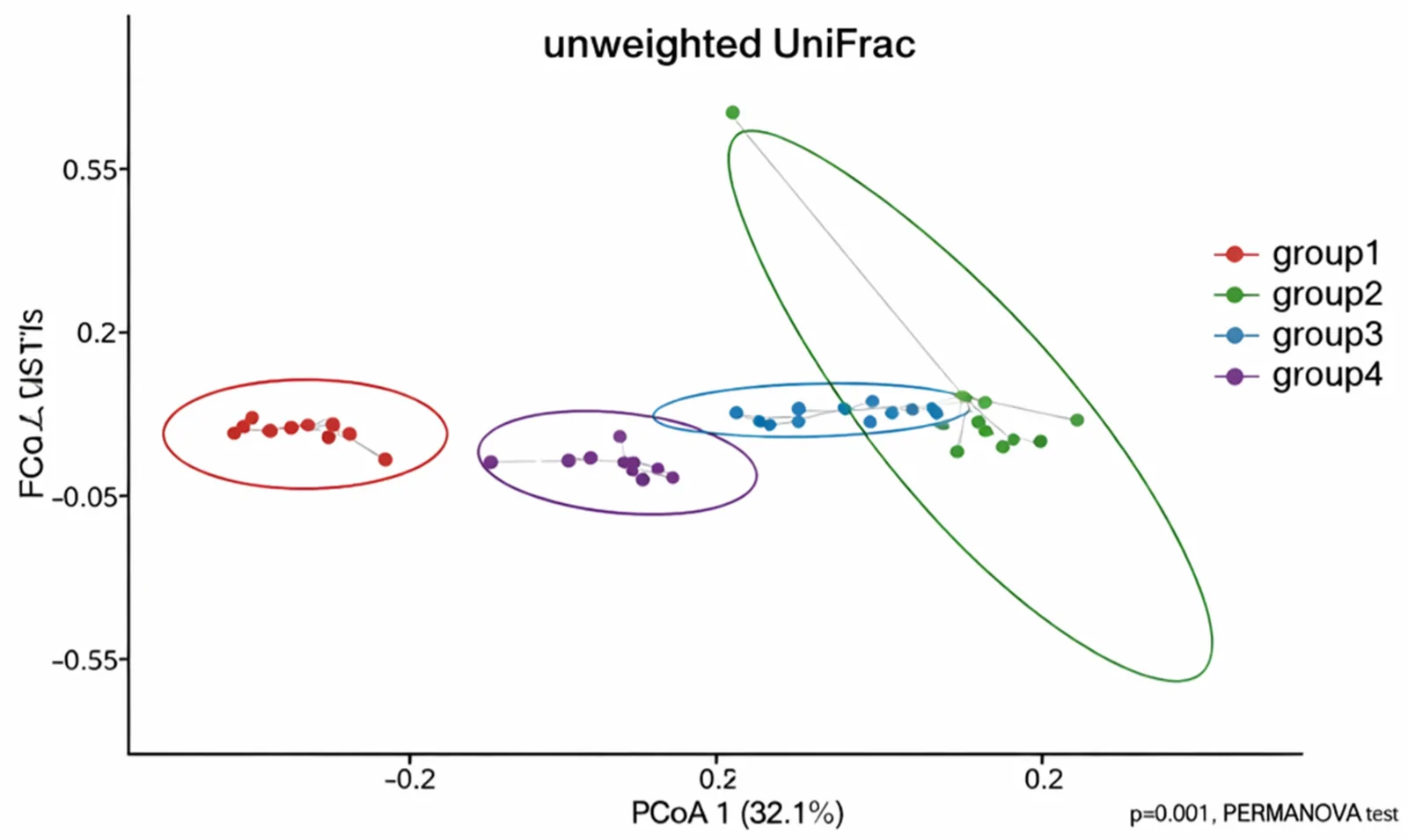
Beta diversity: Group 1: LP NCG− (13% CP); Group 2: LP NCG+ (13% CP + 0.1% NCG); Group 3: HP NCG− (15% CP); Group 4: HP NCG+ (15% CP + 0.1% NCG).

Beta diversity analysis was conducted to determine differences in group flora using the Bray–Curtis genus (Figure 2). HP NCG− (Group 3) shows significant separation from the others, particularly on the vertical axis (PCoA2), indicating it has a distinct flora structure compared to Groups 1, 2, and 4. The beta diversity of Groups 3 and 4 and the unweighted UniFrac microbial distance (Figure 3) were compared to Groups 1 and 2. It was found to be higher compared to the other groups.

#### 3.10.3. Phylum-Level Composition

**Figure 4:**
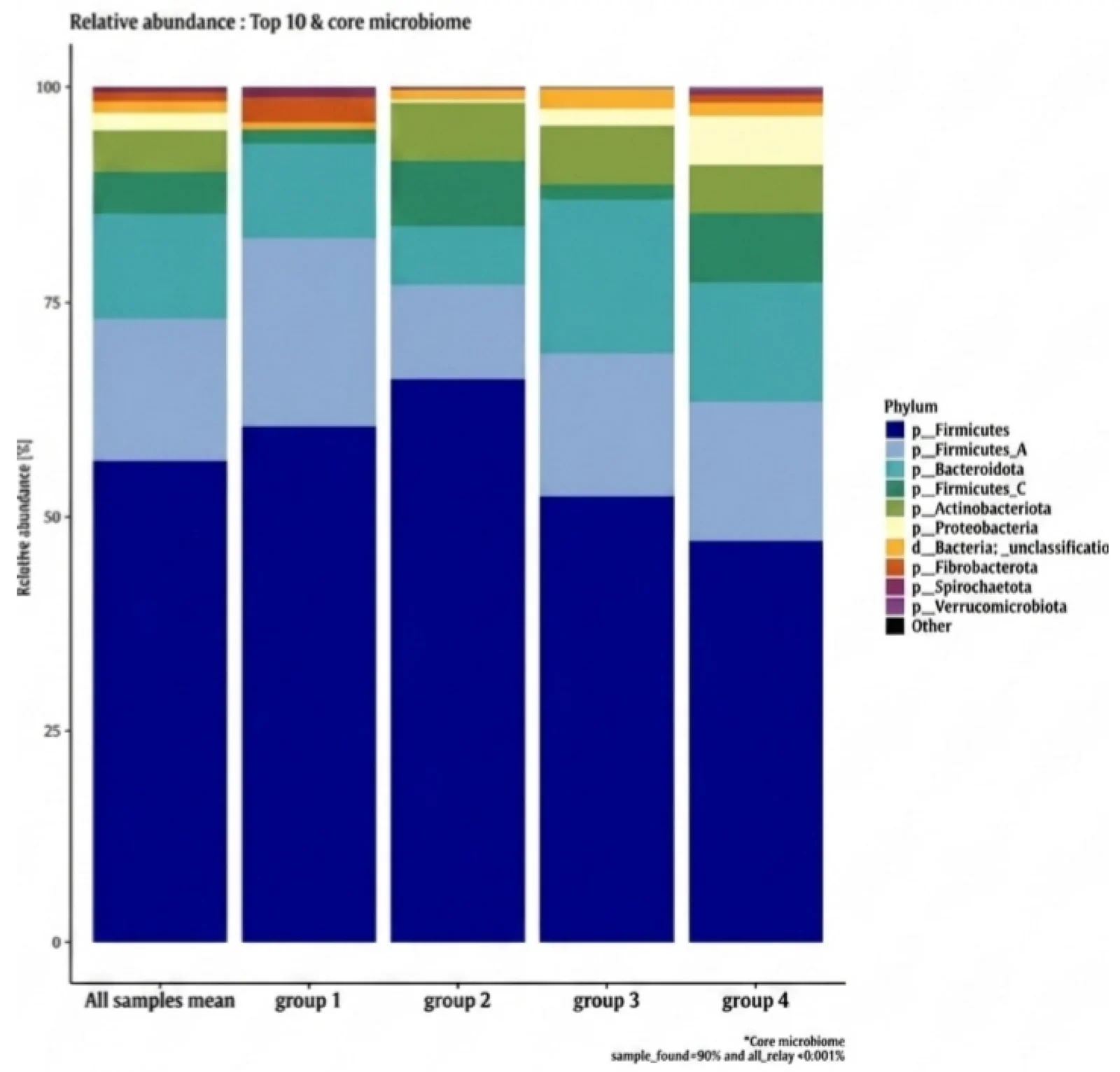
Group 1: LP NCG− (13% CP); Group 2: LP NCG+ (13% CP + 0.1% NCG); Group 3: HP NCG− (15% CP); Group 4: HP NCG+ (15% CP + 0.1% NCG). All sample means. Relative abundance of bacterial composition at the phylum and genus levels. The bacterial composition at the level of fecal matter.

The bacterial composition at both the phylum and genus levels was analyzed to identify specific taxonomic shifts across the groups (Figure 4). The bacterial composition at the level of fecal matter is shown in the figure. The predominant phyla in all groups were Firmicutes and Firmicute A, followed by Bacteroidota. In particular, LP NCG+ (Group 2) pigs showed the highest relative abundance of Firmicutes, and HP NCG− (Group 3) pigs showed the highest relative abundance of Bacteroidota.

**Figure 5:**
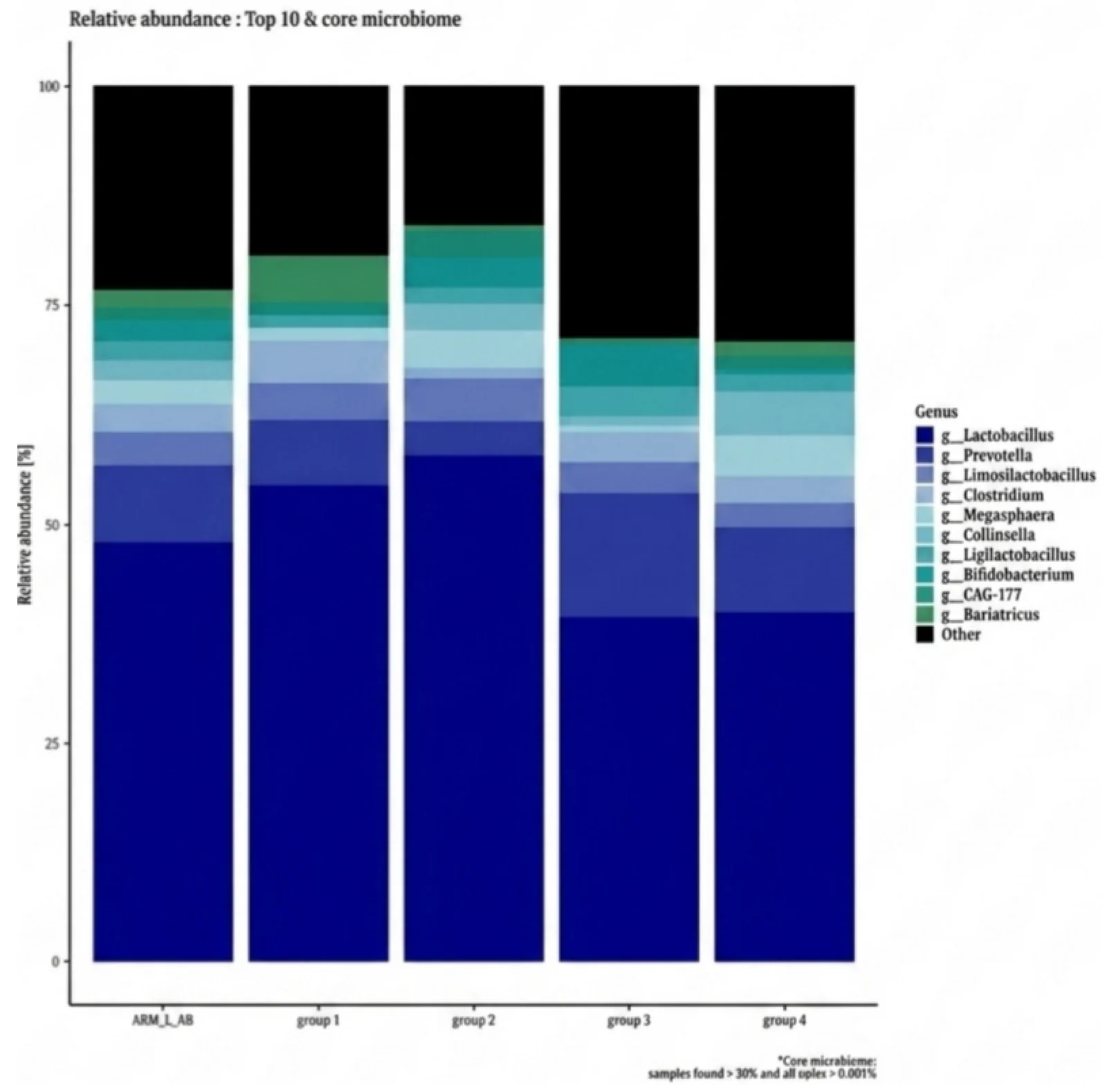
Group 1: LP NCG− (13% CP); Group 2: LP NCG+ (13% CP + 0.1% NCG); Group 3: HP NCG− (15% CP); Group 4: HP NCG+ (15% CP + 0.1% NCG). AEMEAN: All sample means. The bacterial composition at the level of fecal matter.

The dominant genus in all groups was Lactobacillus, followed by Prevotella, Limosilactobacillus, and Clostridium (Figure 5). LP NCG− (Group 1) appears to have the highest relative abundance of *Lactobacillus* compared to the other groups.

**Figure 6:**
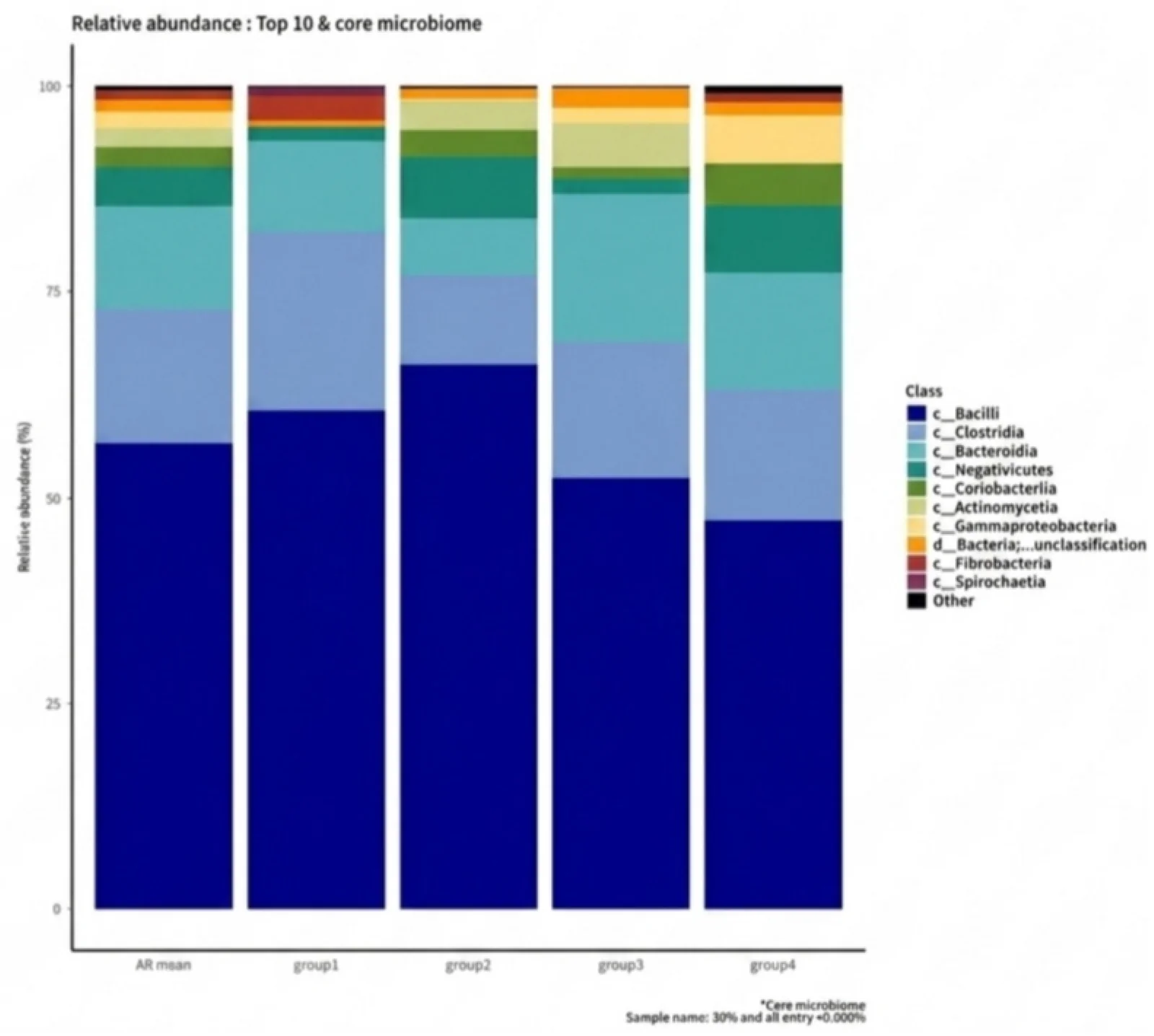
Group 1: LP NCG− (13% CP); Group 2: LP NCG+ (13% CP + 0.1% NCG); Group 3: HP NCG− (15% CP); Group 4: HP NCG+ (15% CP + 0.1% NCG). AEMEAN: All sample means. The bacterial composition at the fecal matter level is shown in the figure.

The bacterial composition of the fecal samples is shown in Figure 6. In all groups, the major classes were Bacilli, Clostridia, and Bacteroidia. High-protein diets of HP NCG− and HP NCG+ (Groups 3 and 4) show a lower relative abundance of *Bacilli* compared to the low-protein groups of LP NCG− and LP NCG+ (Groups 1 and 2). In the high-protein groups, the HP NCG+ group (Group 4) shows a noticeable shift, with a decrease in *Bacilli* and an increase in the diversity/abundance of other classes like *Clostridia* and *Bacteroidia* compared to the HP NCG- group (Group 3). In the low-protein groups, adding NCG (LP NCG+, Group 2) appears to slightly increase the proportion of *Bacilli* compared to the non-supplemented group (Group 1).

**Figure 7:**
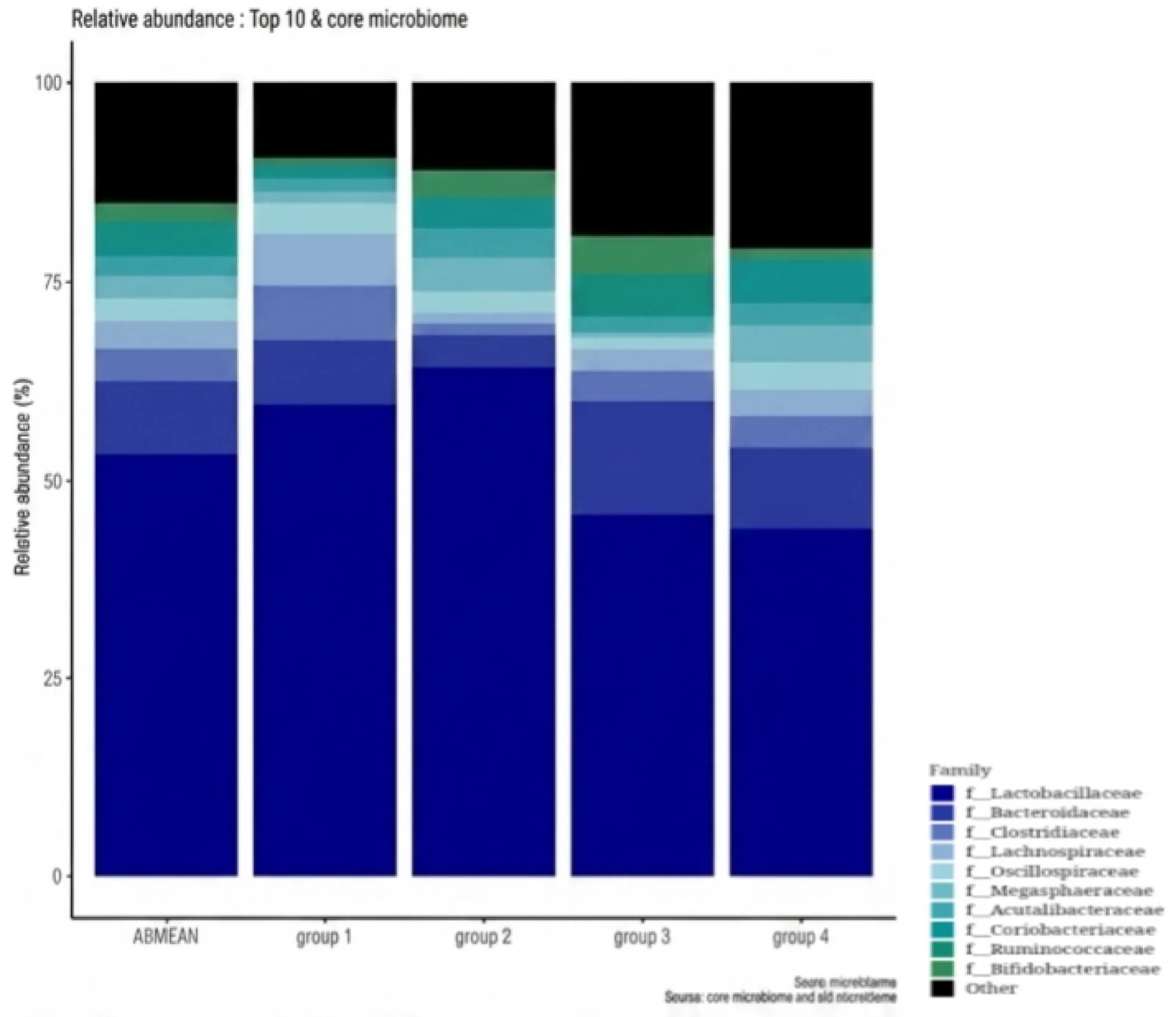
Group 1: LP NCG− (13% CP); Group 2: LP NCG+ (13% CP + 0.1% NCG); Group 3: HP NCG− (15% CP); Group 4: HP NCG+ (15% CP + 0.1% NCG). AEMEAN: All sample means. The bacterial composition at the level of fecal family is shown in the figure.

**Figure 8:**
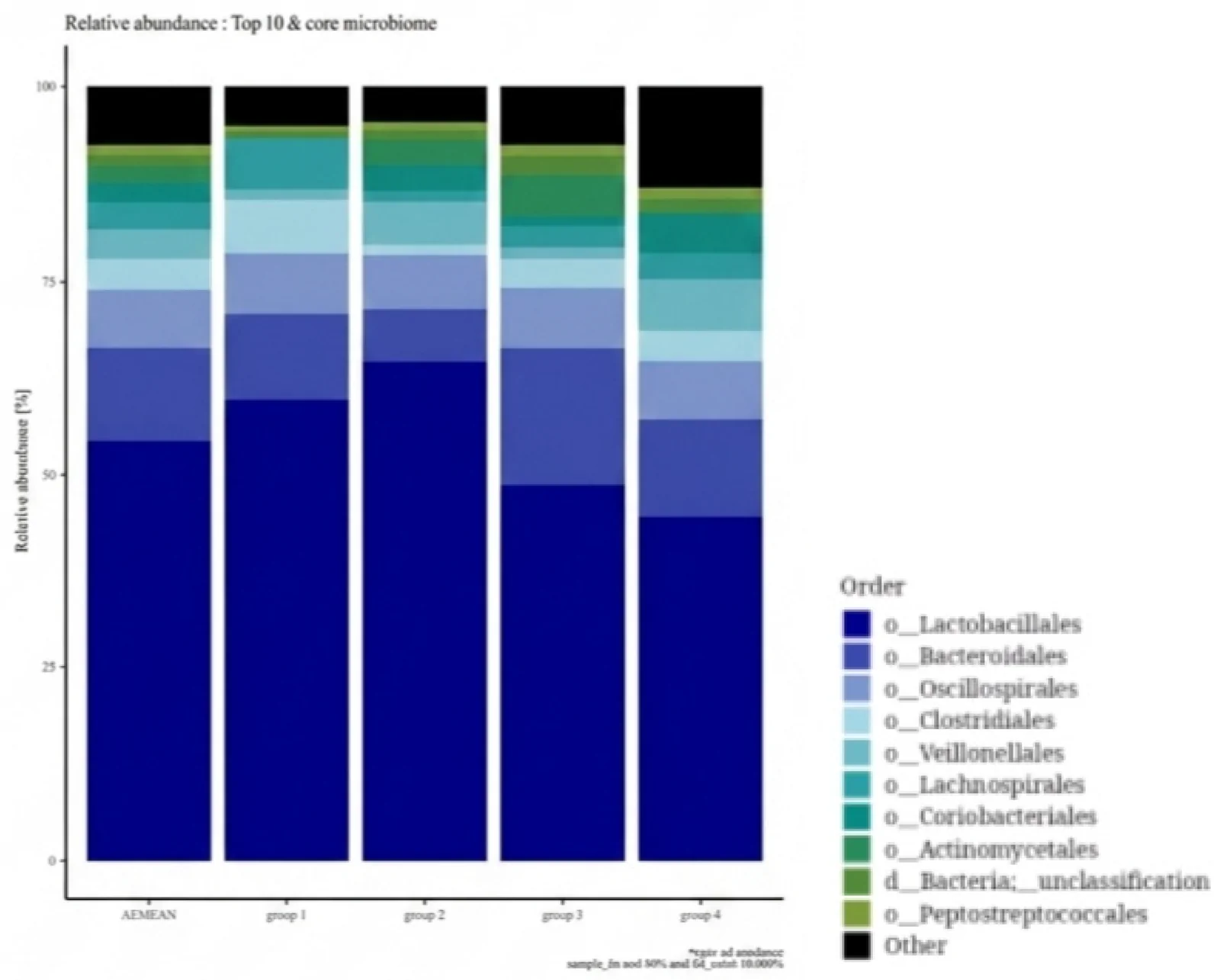
Group 1: LP NCG− (13% CP); Group 2: LP NCG+ (13% CP + 0.1% NCG); Group 3: HP NCG− (15% CP); Group 4: HP NCG+ (15% CP + 0.1% NCG). AEMEAN: All sample means. The bacterial composition at the level of fecal family is shown in the figure.

The bacterial composition at the level of fecal family and order was Figure 7 and Figure 8. The predominant families in all groups were Lactobacillaceae and Bacteroidaceae, followed by Clostridiaceae. Lactobacillaceae showed the highest relative abundance in Group 2 (LP NCG+) compared to other groups.

## 4. Discussion

High-protein diets have primarily been provided, historically and currently, to boost swine farm output. Nevertheless, research is now being conducted to evaluate dietary protein reductions as a way to lessen nitrogen emissions from urine and feces [1]. Pigs fed high-protein diets rather than low-protein diets have more indigestible protein content from the small intestine moving into the large intestine [17]. The direction of the microbiota shift brought on by the high indigestible protein may decrease the hindgut’s consumption of fiber, per the findings of Sung [18]. Dietary protein composition has the power to dramatically alter microflora, which in turn alters how microbes process nutrients [17].

This experiment showed that dietary treatments with two different dietary crude protein densities—low-protein and high-protein, with or without NCG supplementation—had no significant effect on growth performance in finishing pigs. Neither protein density nor NCG supplementation altered ADG, ADFI, or FCR, indicating that growth performance was resilient to these dietary changes. This aligns with previous findings, suggesting that moderate reductions in dietary protein do not impair growth when amino acid requirements are met. Mechanistically, this may reflect the pig’s ability to adapt nitrogen metabolism and maintain feed efficiency despite lower crude protein intake. In the present study, no increase in ADG was observed with high-protein diets. Zhao [19] found that 2% or 4% low dietary protein did not significantly influence the growth performance of growing–finishing pigs. Ye [12] and Zhu [20] observed that 0.1% dietary NCG supplementation had no influence on the growth performance of finishing pigs. In contrast to this study, Li [21] used a low dietary crude protein reduction of 11.14% with 0.0008% NCG supplementation and found an improvement in the growth performance of finishing pigs. Several studies have reported that the correlation between the increase in dietary protein and improved nutrient digestibility could improve growth performance [22]. Reducing dietary CP by up to 14% can reduce ADG and FCR if not adequately equated with amino acids. A lowered-protein diet supplemented with NCG can maintain similar growth performance to a normal-protein diet. However, this study found no increase in ADG with high-protein-density diets, but low-protein and high-protein diets without NCG supplementation showed a tendency for FCR to decrease. The tendency for reduced FCR in pigs fed diets without NCG at week 5 (*p* = 0.0547) suggests a possible transient effect of protein density on feed efficiency, though this did not persist over the full experimental period. The dissimilarity with other studies may be due to the levels of dietary protein, the reduction level of protein, and the NCG level used.

Blood biochemical indices revealed clear protein-dependent effects. Numerous studies have pointed out that BUN concentration reflects nitrogen metabolism, as urea is synthesized in the liver and excreted via the kidneys [23]. Elevated BUN indicates greater protein catabolism and nitrogen excretion [22]. A measure of muscle mass in the body is blood creatinine [1]. In this experiment, BUN, total protein, glucose, and albumin levels decreased with the reduction in dietary protein without NCG, while creatinine levels increased with the reduction in dietary protein and increased as dietary protein levels increased with NCG supplementation. Zhao [19] found that the concentration of serum urea nitrogen (SUN) was considerably reduced by a 2% or 4% decrease in dietary protein. Niyonsaba [4] used CP at 12%, 13%, 14%, 15%, 16%, and 17% for the early stage of finishing and 11%, 12%, 13%, 14%, 15%, and 16% for the later stage of finishing, which linearly increased BUN as dietary CP levels increased. Wang [10] observed that 0.05% and 0.01% NCG supplementation did not influence BUN. As reported by Yan [24], blood total protein cannot be influenced by a 1% difference in dietary protein. Depending on protein level, NCG supplementation can affect muscle protein metabolism and nutrient utilization—such as by changing creatinine but not BUN, total protein, glucose, or albumin levels—compared to without NCG. These differences with other studies may be due to the levels of protein and NCG used and the growth stage of the pigs.

Nutrient digestibility did not differ among treatments, consistent with the lack of improvement in ADG. This suggests that neither protein density nor NCG supplementation enhanced nutrient utilization efficiency in finishing pigs [24]. Niyonsaba [4] used CP at 12%, 13%, 14%, 15%, 16%, and 17% for the early stage of finishing and 11%, 12%, 13%, 14%, 15%, and 16% for the later stage of finishing, and no significant effect was noticed. However, Wang [25] reported that a decrease in dietary protein with or without NCG supplementation decreased nutrient digestibility, the same as O’Connell [26] in finishing pigs. Several studies have reported an increase in the ATTD of CP and improved growth performance with increased protein density. The non-significance of ADG in this study may be due to a lack of increased nutrient digestibility in the treatment groups, and NCG addition did not reverse or increase digestibility.

Numerous analyses of nitrogen efficiency in pigs with varying dietary CP amounts have been conducted; the majority of the findings indicate that a 4-percentage-point-or-lower reduction in dietary protein content has no discernible effect on nitrogen deposition [17]. In this experiment, the dietary crude protein level had an influence on dry matter intake and nitrogen intake. Nutrient digestibility and nitrogen retention were largely unaffected by NCG, but higher protein diets increased dry matter and nitrogen intake. Protein density primarily drives nitrogen utilization, while NCG plays a limited role in modulating digestibility. Zhao [19] reported that at a reduction of 2% and 4% dietary crude protein levels, the nitrogen intakes were lower by 11.6% and 26.1% at the 4% protein level than at the 2% protein level. Also, total nitrogen excretion at 2% and 4% lower protein levels was reduced by 16.9% and 31.9%. Wang [25] reported no significant effect of three dietary protein densities—very low at 12%, medium–low at 15%, and high protein at 18%— in diets fed to pigs. Furthermore, it was reported that pigs fed high-protein diets have greater nitrogen intake, urinary N, and total N losses than other levels, and that pigs fed medium–low protein and very low protein have greater N retention-to-intake ratios than pigs fed high protein, and no observable difference between pigs fed medium–low protein and very low protein. These differences may be due to the level of dietary protein.

Gas emissions are caused by the non-digestion of protein. Different studies have reported improvements in nutrient digestibility by providing high-nutrient/protein diets, helping to reduce gas emissions by reducing substrates for microbial fermentation in the colon [22]. There was no difference in noxious gas emissions between groups. Similar to our study, Zhao [19] reported no significant effect of decreasing dietary protein levels by 2% and 4% on NH_3_-N contents. Niyonsaba [4] observed a linear effect of increasing CP levels on NH_3_ and hydrogen sulfide in gaseous emissions in growing–finishing pigs using 14–19% dietary protein. N excretion was lowered by reducing the crude protein content of pig diets and keeping the amount of lysine available at 8.2 g/kg. This, in turn, reduced N losses as NH_3_ and N levels were reduced in slurry [27]. The non-significance of gas emissions in this study may be due to the lack of influence on nutrients. Although there was no effect on noxious gas in this experiment, NCG has been known to act as a regulator, reducing harmful nitrogenous waste that contributes to odor.

There was no difference in backfat thickness or LMP between groups. Zhao [19] reported no influence of dietary protein levels on backfat thickness. In contrast to this result, Wang [10] observed higher LMP with pigs fed 0.05% supplementation of NCG. It is highly likely that the carcass traits of finishing pigs are more sensitive to the presence or absence of specific metabolic regulators like NCG.

N-carbamylglutamate supplementation has been shown in different experiments to improve the quality of the meat produced by finishing pigs on a low-protein diet [23]. In agreement with this study, Wang [10] reported that 0.1% NCG supplementation could be beneficial for meat quality, and Zhu [20] observed an increase in longissimus dorsi muscle area with 0.1% NCG supplementation in growing–finishing pigs. Wang [10] reported that 0.05% NCG supplementation significantly influenced the increase in cooking holding percentage but had no effect on other qualities. Wang [28] observed an improvement in the meat quality of finishing pigs with low protein density in a 60-day experiment. NCG promotes loin development and water-holding capacity more efficiently than simply increasing protein content and is highly valuable as a key additive for preserving or enhancing carcass value, especially when feeding low-protein feed.

There was no difference on carcass quality between groups. Dietary protein levels alone ranging from 11% to 17% do not significantly alter carcass quality, as evidenced by our results and the findings of Zhao [19] and Niyonsaba [4]. However, the inclusion of 0.1% NCG acts as a crucial compensatory factor. As reported by Ye [12], Zhu [20], and Wang [10], NCG supplementation improves carcass weight and traits even under low-protein regimens. These results suggest that NCG supplementation in low-protein diets is a superior strategy for enhancing carcass value compared to simply increasing dietary protein concentrations. Carcass traits such as backfat thickness, lean meat percentage, and carcass grade were not influenced by treatments, indicating that protein density and NCG supplementation did not compromise carcass yield. However, meat quality parameters revealed notable effects of NCG. Longissimus muscle area and cooking loss were significantly increased in LP NCG+ pigs compared to HP NCG+, suggesting that NCG may enhance muscle development and alter water-holding properties, particularly under reduced protein conditions. These indicate a potential role for NCG in improving pork quality, even when growth performance remains unaffected.

NCG supplementation and dietary protein levels exert distinct yet complementary effects on amino acid metabolism and intestinal fermentation in finishing pigs. In this experiment, the dietary crude protein level had an influence on the essential amino acids isoleucine, histidine, and lysine and the non-essential amino acids alanine and tyrosine. This underscores the importance of dietary protein in maintaining carcass amino acid profiles, while NCG supplementation did not exert any significant effects. NCG (0.05–0.1%) significantly elevates plasma arginine and other free amino acids (Wang [10]), enhancing protein utilization efficiency. Concurrently, dietary protein levels modulate core metabolic pathways, including the citrate cycle and pyruvate metabolism, while influencing the cecal concentrations of branched-chain fatty acids (BCFAs) (Zhou [29]). Our experimental results on this indicate that NCG acts as a metabolic enhancer that can mitigate the physiological shifts caused by low-protein diets, effectively maintaining systemic amino acid balance and metabolic homeostasis.

Higher microbial diversity is generally associated with better gut health and stronger immune resilience in pigs [14]. Thus, supplementing both high-protein and low-protein diets with NCG improves species diversity in the gut microbiome of pigs. In high-protein diets, when a large amount of protein passes into the large intestine, proteolytic bacteria like *Clostridium* produce harmful metabolites like ammonia and amines, while low dietary crude protein increases *Lactobacillus* and decreases harmful bacteria. This indicates that while dietary protein density remains the primary determinant of metabolic and carcass outcomes, NCG supplementation exerts beneficial effects on meat quality and microbial diversity. Future studies should explore the mechanistic basis of NCG’s influence on muscle metabolism and gut microbiota, and whether these changes translate into long-term improvements in production efficiency and pork quality. Wang [28] observed an improvement in the structure of the gut microbiota of finishing pigs with low protein density in a 60-day experiment. Zhou [29] reported that the relative abundance of *Prevotella* in the cecum was significantly higher in the low-protein group than in the normal-protein group. *Lactobacillus* is often considered beneficial bacteria that help maintain gut balance and support the immune system. Zhou [29] reported that the relative abundance of Lactobacillus in the colon was significantly reduced by the low-protein diet, according to pyrosequencing of the V1–V3 region of the 16S rRNA genes. These findings imply that a low-protein diet may alter the microbial makeup and metabolites of the hindgut without influencing the pigs’ ability to grow; nevertheless, the possible effects of this are yet unclear. NCG has been reported to act as a modulator, decreasing harmful nitrogenous waste that contributes to odor while supporting favorable bacterial populations in the gut [30,31]. The observed shifts in microbiota composition suggest that reducing dietary protein with NCG supplementation may optimize the gut environment. This shift likely mitigates the negative impacts of undigested protein fermentation, such as the production of toxic metabolites, thereby supporting the pig’s overall metabolic efficiency even under low-protein conditions. These differences may be due to the level of dietary protein, NCG dosage, and pig breeds used.

## 5. Conclusions

Low-protein diets supplemented with 0.1% N-carbamylglutamate (NCG) maintained growth performance while improving nitrogen retention, blood profile, carcass amino acid composition, meat quality, and gut microbiota in finishing pigs. Importantly, reducing dietary protein to 13% did not compromise digestibility or carcass yield, highlighting NCG’s role as a compensatory additive under protein-restricted conditions. These findings support the use of NCG to enhance metabolic efficiency and pork quality while addressing environmental concerns through reduced nitrogen excretion. Future work should investigate the interaction between NCG and limiting amino acids such as arginine to refine low-protein feeding strategies.

## Figures and Tables

**Table 1:** Composition of finishing pig diets.

Item	Experimental Diet	
	High Crude Protein (Basal Diet)	Low Crude Protein (Basal Diet—2% CP)
Ingredients (%)		
Corn	71.02	75.92
Soybean meal	13.06	7.65
DDGS	10.00	10.00
Tallow	2.65	2.74
MDCP	1.10	1.27
Limestone	0.73	0.64
Salt	0.30	0.30
Methionine (99%)	0.05	0.08
Lysine (78%)	0.50	0.69
Threonine (99%)	0.11	0.20
Tryptophan (99%)	0.05	0.08
Mineral mix ^1^	0.20	0.20
Vitamin mix ^2^	0.20	0.20
Choline (25%)	0.03	0.03
Total	100.00	100.00
Calculated value		
Crude protein, %	15.00	13.00
Calcium, %	0.60	0.60
Phosphorus, %	0.55	0.55
Lysine, %	1.00	1.00
Methionine, %	0.30	0.30
Metabolizable energy, kcal/kg	3300	3300
Fat, %	6.14	6.31

^1^ Provided per kg diet: Fe, 115 mg as ferrous sulfate; Cu, 70 mg as copper sulfate; Mn, 20 mg as manganese oxide; Zn, 60 mg as zinc oxide; I, 0.5 mg as potassium iodide; and Se, 0.3 mg as sodium selenite. ^2^ Provided per kg of diet: vitamin A, 13,000 IU; vitamin D_3_, 1700 IU; vitamin E, 60 IU; vitamin K_3_, 5 mg; vitamin B_1_, 4.2 mg; vitamin B_2_, 19 mg; vitamin B_6_, 6.7 mg; vitamin B_12_, 0.05 mg; biotin, 0.34 mg; folic acid, 2.1 mg; niacin, 55 mg; D-calcium pantothenate, 45 mg.

**Table 2:** The effect of dietary NCG supplementation with different crude protein densities on growth performance in finishing pigs ^1^.

Items	LP		HP		SEM ^2^	*p*-Value		
	NCG−	NCG+	NCG−	NCG+		CP Effect	NCG Effect	Interaction
Body weight, kg								
Initial	55.08	55.07	55.07	55.07	1.03	0.9973	0.9996	0.9942
Week 5	82.35	83.05	83.66	84.13	1.50	0.4294	0.6988	0.9405
Week 10	112.81	114.94	115.8	117.14	2.18	0.2415	0.4322	0.8560
Initial–Week 5								
ADG, g	779	799	817	830	19	0.0765	0.3797	0.8557
ADFI, g	2081	2125	2150	2170	36	0.1254	0.3921	0.7489
FCR	2.673	2.663	2.638	2.615	0.021	0.0547	0.4471	0.7581
Week 5–Week 10								
ADG, g	870	911	918	943	23	0.0964	0.1713	0.7303
ADFI, g	2622	2692	2727	2770	59	0.1312	0.3435	0.8197
FCR	3.017	2.959	2.973	2.944	0.034	0.4261	0.2448	0.7011
Overall								
ADG, g	825	855	868	887	21	0.0819	0.2447	0.7834
ADFI, g	2352	2409	2438	2470	47	0.1201	0.3495	0.7891
FCR	2.854	2.820	2.815	2.789	0.025	0.1760	0.2446	0.8800

^1^ Abbreviation: LP NCG−, low-protein NCG−, basal diet: 13% CP; LP NCG+, low-protein NCG+, basal diet: 13% CP + NCG 0.1% (1 kgton); HP NCG−, high-protein NCG−, basal diet: 15% CP; HP NCG+, high-protein NCG+, basal diet: 15% CP + NCG 0.1% (1 kg/ton). ^2^ Standard error of means.

**Table 3:** The effect of dietary NCG supplementation with different crude protein densities on blood profile in finishing pigs ^1^.

Items	LP		HP		SEM ^2^	*p*-Value		
	NCG−	NCG+	NCG−	NCG+		CP Effect	NCG Effect	Interaction
Finish								
BUN, mg/dL	10.0 ^b^	9.3 ^ab^	11.5 ^a^	11.3 ^ab^	0.4	0.0005	0.1992	0.5098
Creatinine, mg/dL	1.13 ^ab^	1.34 ^a^	1.15 ^ab^	1.31 ^b^	0.1	0.9517	0.0113	0.7022
Total protein, g/dL	6.88 ^b^	6.78 ^b^	7.73 ^a^	7.80 ^a^	0.2	0.0013	0.9565	0.7037
Albumin, g/dL	3.45 ^b^	3.48 ^ab^	4.60 ^a^	4.73 ^ab^	0.1	<0.0001	0.5025	0.6531
Glucose, mg/dL	79.3 ^b^	84.8 ^ab^	87.5 ^a^	90.0 ^ab^	2	0.0057	0.0702	0.4705
Triglycerides, mg/dL	29.0	30.3	31.8	32.5	3.5	0.4903	0.7808	0.9444
Cholesterol, mg/dL	94.5	96.3	98.0	99.3	4.9	0.5215	0.7657	0.9603

^1^ Abbreviation: LP NCG−, low-protein NCG−, basal diet: 13% CP; LP NCG+, low-protein NCG+, basal diet: 13% CP + NCG 0.1% (1 kgton); HP NCG−, high-protein NCG−, basal diet: 15% CP; HP NCG+, high-protein NCG+, basal diet: 15% CP + NCG 0.1% (1 kg/ton). ^2^ Standard error of means. ^a,b^ Means in the same row with different superscripts differ significantly (*p* < 0.05).

**Table 4:** The effect of dietary NCG supplementation with different crude protein densities on nutrient digestibility in finishing pigs ^1^.

Items, %	LP		HP		SEM ^2^	*p*-Value		
	NCG−	NCG+	NCG−	NCG+		CP Effect	NCG Effect	Interaction
Finish								
Dry matter	73.83	73.90	74.44	74.01	0.51	0.4916	0.7299	0.6330
Nitrogen	68.28	70.14	71.66	72.03	1.83	0.1692	0.563	0.7047
Energy	72.97	72.66	73.01	73.33	0.53	0.5091	0.9908	0.5625

^1^ Abbreviation: LP NCG−, low-protein NCG−, basal diet: 13% CP; LP NCG+, low-protein NCG+, basal diet: 13% CP + NCG 0.1% (1 kgton); HP NCG−, high-protein NCG−, basal diet: 15% CP; HP NCG+, high-protein NCG+, basal diet: 15% CP + NCG 0.1% (1 kg/ton). ^2^ Standard error of means.

**Table 5:** The effect of dietary NCG supplementation with different crude protein densities on N retention in finishing pigs ^1^.

Items	LP		HP		SEM ^2^	*p*-Value		
	NCG−	NCG+	NCG−	NCG+		CP Effect	NCG Effect	Interaction
Finish								
DM intake, g/d	2247.9 ^b^	2278.8 ^ab^	2356.3 ^a^	2381.6 ^ab^	46.10	0.0410	0.5549	0.9534
N intake, g/d	63.31 ^b^	64.14 ^ab^	65.84 ^a^	66.94 ^ab^	1.08	0.0310	0.3864	0.9102
Fecal N excretion, g/d	24.58	24.40	24.98	24.83	1.01	0.6897	0.8747	0.9903
N digestion, g/d	38.74	39.74	40.86	42.11	1.21	0.0904	0.3676	0.9277
N retention rate, %	61.13	61.92	62.10	62.94	1.47	0.5153	0.5913	0.9934

^1^ Abbreviation: LP NCG−, low-protein NCG−, basal diet: 13% CP; LP NCG+, low-protein NCG+, basal diet: 13% CP + NCG 0.1% (1 kgton); HP NCG−, high-protein NCG−, basal diet: 15% CP; HP NCG+, high-protein NCG+, basal diet: 15% CP + NCG 0.1% (1 kg/ton). ^2^ Standard error of means. ^a,b^ Means in the same row with different superscripts differ significantly (*p* < 0.05).

**Table 6:** The effect of dietary NCG supplementation with different crude protein on gas emission in finishing pigs ^1^.

Items, ppm	LP		HP		SEM ^2^	*p*-Value		
	NCG−	NCG+	NCG−	NCG+		CP Effect	NCG Effect	Interaction
Finish								
NH_3_	10.75	10.13	12.25	11.50	0.83	0.1071	0.4211	0.9409
H_2_S	7.15	7.30	7.83	7.63	0.50	0.3332	0.9606	0.7303
Methyl mercaptans	8.88	8.38	8.80	8.63	0.72	0.9046	0.6454	0.8241
Acetic acid	11.25	11.38	11.75	11.70	0.55	0.4639	0.9463	0.8752
CO_2_	14,600	14,500	14,850	15,000	342.00	0.2950	0.9430	0.7215

^1^ Abbreviation: LP NCG−, low-protein NCG−, basal diet: 13% CP; LP NCG+, low-protein NCG+, basal diet: 13% CP + NCG 0.1% (1 kgton); HP NCG−, high-protein NCG−, basal diet: 15% CP; HP NCG+, high-protein NCG+, basal diet: 15% CP + NCG 0.1% (1 kg/ton). ^2^ Standard error of means.

**Table 7:** The effect of dietary NCG supplementation with different crude protein densities on backfat thickness and LMP in finishing pigs ^1^.

Items	LP		HP		SEM ^2^	*p*-Value		
	NCG−	NCG+	NCG−	NCG+		CP Effect	NCG Effect	Interaction
Finish								
Backfat thickness, mm	19.23	19.81	19.87	20.34	0.56	0.3015	0.3531	0.9220
LMP, %	51.48	51.75	51.86	52.27	0.53	0.3923	0.5279	0.9023

^1^ Abbreviation: LP NCG−, low-protein NCG−, basal diet: 13% CP; LP NCG+, low-protein NCG+, basal diet: 13% CP + NCG 0.1% (1 kgton); HP NCG−, high-protein NCG−, basal diet: 15% CP; HP NCG+, high-protein NCG+, basal diet: 15% CP + NCG 0.1% (1 kg/ton). ^2^ Standard error of means.

**Table 8:** The effect of dietary NCG supplementation with different crude protein densities on meat quality in finishing pigs ^1^.

Items	LP		HP		SEM ^2^	*p*-Value		
	NCG−	NCG+	NCG−	NCG+		CP Effect	NCG Effect	Interaction
pH	5.20	5.25	5.22	5.28	0.05	0.6095	0.2923	0.8776
Longissimus muscle area, mm^2^	7431.17 ^ab^	7825.88 ^b^	7464.91 ^ab^	7842.31 ^a^	41.20	0.5540	<0.0001	0.8372
Water-holding capacity, %	46.58	47.85	46.90	48.26	2.60	0.8908	0.6224	0.9865
Meat color								
L*	49.78	49.53	49.40	49.24	0.91	0.7178	0.826	0.9615
a*	14.94	14.70	14.86	14.71	0.48	0.9388	0.6869	0.9347
b*	6.01	6.14	6.15	6.09	0.20	0.8339	0.8715	0.6458
Cooking loss, %	32.23 ^ab^	29.89 ^a^	32.73 ^ab^	29.81 ^b^	0.54	0.7119	0.0004	0.5993
Drip loss, %								
d1	4.34	4.29	4.37	4.27	0.27	0.9783	0.7787	0.9421
d3	11.55	11.46	11.59	11.41	0.37	0.9763	0.7249	0.9029
d5	16.77	16.63	16.80	16.56	0.40	0.9587	0.6467	0.9005
d7	22.67	22.45	22.68	22.36	0.53	0.9412	0.6203	0.9375
Sensory evaluation								
Color	3.25	3.00	3.00	3.13	0.10	0.5250	0.5250	0.0731
Marbling	3.50	3.88	3.88	3.75	0.33	0.7087	0.7087	0.4590
Firmness	3.25	3.25	3.13	3.00	0.37	0.6245	0.8698	0.8698

^1^ Abbreviation: LP NCG−, low-protein NCG−, basal diet: 13% CP; LP NCG+, low-protein NCG+, basal diet: 13% CP + NCG 0.1% (1 kgton); HP NCG−, high-protein NCG−, basal diet: 15% CP; HP NCG+, high-protein NCG+, basal diet: 15% CP + NCG 0.1% (1 kg/ton). ^2^ Standard error of means. ^a,b^ Means in the same row with different superscripts differ significantly (*p* < 0.05).

**Table 9:** The effect of dietary NCG supplementation with different crude protein densities on carcass grade and backfat thickness in finishing pigs ^1^.

Items	LP		HP		SEM ^2^	*p*-Value		
	NCG−	NCG+	NCG−	NCG+		CP Effect	NCG Effect	Interaction
Carcass weight, kg	90.27	90.52	89.38	90.53	6.01	0.5934	0.5305	0.8424
Backfat thickness, mm	21.71	21.86	21.79	20.91	2.00	0.5001	0.9785	0.9968
1+, %	25.00	27.50	30.00	25.00	-	-	-	-
1, %	42.50	40.00	40.00	42.50	-	-	-	-
2, %	32.50	32.50	30.00	32.50	-	-	-	-

^1^ Abbreviation: LP NCG−, low-protein NCG−, basal diet: 13% CP; LP NCG+, low-protein NCG+, basal diet: 13% CP + NCG 0.1% (1 kgton); HP NCG−, high-protein NCG−, basal diet: 16% CP; HP NCG+, high-protein NCG+, basal diet: 16% CP + NCG 0.1% (1 kg/ton). ^2^ Standard error of means.

**Table 10:** The effect of dietary NCG supplementation with different crude protein densities on carcass amino acid content in finishing pigs ^1^.

Items, %	LP		HP		SEM ^2^	*p*-Value ^3^		
	NCG−	NCG+	NCG−	NCG+		CP Effect	NCG Effect	Interaction
Essential amino acid								
Threonine	0.80	0.87	0.91	0.96	0.07	0.1927	0.4670	0.8777
Valine	1.02	1.08	1.10	1.13	0.05	0.2470	0.3827	0.7806
Isoleucine	0.59 ^b^	0.63 ^ab^	0.87 ^a^	0.90 ^ab^	0.10	0.0180	0.7641	0.9329
Leucine	1.43	1.52	1.55	1.61	0.08	0.2429	0.3865	0.8146
Phenylalanine	0.51	0.57	0.64	0.66	0.07	0.1711	0.5980	0.7911
Histidine	1.12 ^b^	1.21 ^ab^	1.29 ^a^	1.33 ^ab^	0.06	0.0420	0.3327	0.7423
Lysine	1.60 ^b^	1.67 ^ab^	1.75 ^a^	1.78 ^ab^	0.05	0.0365	0.3903	0.6719
Arginine	1.39	1.57	1.54	1.59	0.05	0.1079	0.0328	0.2463
Methionine	0.82	0.89	0.93	0.96	0.06	0.1091	0.3973	0.7091
Tryptophan	0.36	0.39	0.46	0.48	0.06	0.1300	0.7445	0.9674
TEAA ^1^	0.96	1.04	1.10	1.14	0.05	0.2041	0.7299	0.9816
Non-essential amino acid								
Aspartic acid	2.00	2.02	2.07	2.09	0.04	0.3396	0.4910	0.9335
Serine	0.74	0.76	0.78	0.81	0.09	0.3127	0.5944	0.8652
Glutamic acid	3.43	3.50	3.55	3.58	0.07	0.1557	0.6576	0.8530
Proline	0.71	0.75	0.82	0.84	0.12	0.4350	0.7822	0.9370
Glysine	0.83	0.86	0.92	0.97	0.04	0.2032	0.5137	0.7200
Alanine	1.03 ^b^	1.07 ^ab^	1.10 ^a^	1.11 ^ab^	0.05	0.0414	0.5478	0.8523
Tyrosine	0.70 ^b^	0.75 ^ab^	0.83 ^a^	0.86 ^ab^	0.05	0.0436	0.2981	0.5197
Cystine	0.34	0.36	0.42	0.50	0.06	0.0665	0.3947	0.7424
TNEAA ^1^	1.22	1.26	1.31	1.34	0.05	0.0722	0.4680	0.9783
Total Amino Acid	1.08	1.14	1.20	1.23	0.05	0.0536	0.3771	0.8032

^1^ Abbreviation: LP NCG−, low-protein NCG−, basal diet: 13% CP; LP NCG+, low-protein NCG+, basal diet: 13% CP + NCG 0.1% (1 kgton); HP NCG−, high-protein NCG−, basal diet: 16% CP; HP NCG+, high-protein NCG+, basal diet: 16% CP + NCG 0.1% (1 kg/ton). TEAA, total essential amino acid; NTEAA, non-total essential amino acid. ^2^ Standard error of means. ^3^ Means in the same row with different superscripts differ significantly (*p* < 0.05). ^a,b^ Means in the same row with different superscript differ significantly (*p* < 0.05).

## Data Availability

Data are available on request due to privacy.
